# Prevalence and associated factors for pterygium in Han and Mongolian adults: a cross-sectional study in inner Mongolian, China

**DOI:** 10.1186/s12886-020-1324-6

**Published:** 2020-02-03

**Authors:** Yuhan Wang, Guangliang Shan, Linyang Gan, Yonggang Qian, Ting Chen, Hailing Wang, Xiaodan Pan, Wenrui Wang, Li Pan, Xia Zhang, Meng Wang, Jin Ma, Yong Zhong

**Affiliations:** 1 Department of Ophthalmology, Peking Union Medical College Hospital, Chinese Academy of Medical Sciences & Peking Union Medical College, No. 1 Shuaifu Yuan, Dongcheng District, Beijing, 100730 China; 2 Department of Epidemiology and Statistics, Institute of Basic Medical Sciences, Chinese Academy of Medical Sciences & School of Basic Medicine, Peking Union Medical College, Beijing, China; 3 Inner Mongolia Center for Disease Control and Prevention, Hohhot, Inner Mongolia Autonomous Region China; 4 School of Medicine, Tsinghua University, Beijing, China

**Keywords:** Pterygium, Prevalence, Han and Mongolian, Risk factors, Protective factors

## Abstract

**Background:**

To investigate the prevalence of pterygium and associated factors in Han and Mongolian adults at four survey sites in Inner Mongolia, China.

**Methods:**

We conducted a population-based, cross-sectional study as part of the China National Health Survey (CNHS). By means of a stratified sampling method, we finally included 2651 participants of 30 years of age or older from a total of 3468 eligible residents. Factors associated with pterygium were analysed by a univariate analysis and logistic regression models.

**Results:**

The study population included 1910 Han and 741 Mongolian adults. The mean age ± standard deviation of the study cohort was 48.93 ± 11.06 years. The overall prevalence of pterygium was 6.4% (*n* = 169); 1.4% (*n* = 38) of the cases were bilateral and 4.8% (*n* = 128) were unilateral. The most common grade of pterygium was Grade 2. Based on the results of the univariate analysis, eleven factors were included in a multivariate analysis. The results indicated that age (*P* < 0.001), outdoor occupation (*P* = 0.026), and time spent in rural areas (*P* < 0.001) were significantly associated with pterygium. Sex and ethnicity were not identified as risk factors.

**Conclusions:**

Our results indicated that outdoor occupation, old age and more time spent in rural areas were risk factors for pterygium in Inner Mongolia. At the same time, town as a survey site (Hohhot and Tsining District) was a protective factor for pterygium. Ethnicity, gender, smoking, diabetes and high blood pressure are not associated with pterygium.

## Background

Pterygium is a proliferative fibrovascular tissue overgrowth arising from bulbar conjunctiva and encroaching onto the cornea. As the disease develops, it can induce severe astigmatism and poor vision. A systematic review [1] of 68 articles with a total of 415,911 participants in 24 countries in 2018 reported that the overall prevalence of pterygium was 12%. A review of the previous literature shows that the prevalence of pterygium varies widely in different regions and ethnic groups, with rates as low as 1.1% [2] and as high as 39.0% [3]. Most epidemiological surveys investigating Chinese pterygium have analysed a single-ethnic group, and have not compared different ethnicities. The prevalence of pterygium in remote areas of China is relatively high. For instance, the prevalence of pterygium was 11.95% in Xinjiang [4], 14.49% in Tibetans in Zeku County, Qinghai Province [5], 17.9% in Mongolians in Henan Mongolian Autonomous County, Qinghai Province [3], and 39.0% in the Bai ethnic group in Dali [6].

There are many risk factors for pterygium, including exposure to ultraviolet radiation, geographic latitude, ageing, nationality and skin colour. The most widely established factor is ultraviolet radiation. The intensity and time of exposure to ultraviolet radiation are significantly correlated with the prevalence [4, 5]. At the same time, some studies have found that the geographical latitude is related to pterygium; for example, the “pterygium belt”, which is located between the latitudes of 37°N and 37°S, has a high prevalence of pterygium [7]. With increasing cultural and trade exchanges between countries, we should also consider the impact of population migration. According to a study of Riau Islanders in 2006 [8], people who had lived on the island since childhood had a higher prevalence of pterygium than foreigners. However, even among those who live in the same fixed environment, the genetic heterogeneity of different races and ethnic groups may affect the prevalence of pterygium. In 2014, our previous survey in Xinjiang [4] showed that the prevalence of pterygium in the Uygur population was lower than that of the Han population living in the same area. Meanwhile, there are also factors that protect against pterygium. For example, it was demonstrated that the wearing of hats and sunglasses could reduce the prevalence of pterygium in Shandong, Yunnan and Ethiopia [9–11].

The China National Health Survey (CNHS) is a nationally representative population-based cross-sectional study conducted in various provinces in China [12]. We selected the Inner Mongolia Autonomous Region as a survey province as a part of the CNHS. Inner Mongolia is located in the northern part of China (longitude, 97°-126° east; latitude 37°-53°, north). According to the results of the China Population Census 2010, people of the Han and Mongolian ethnicities account for 79 and 17% of the total population, respectively, and are the major ethnicities in the region. Through the study of the CNHS in Inner Mongolia Autonomous Region, differences in disease prevalence have been found between people of the Han and Mongolian ethnicities. For example, the prevalence of hypertension and obesity is significantly higher in Mongolians than in Han [13]. In this study, we compared various demographic characteristics between Han and Mongolians in four regions of Inner Mongolia Autonomous Region, China, to assess the prevalence of pterygium and associated factors.

## Methods

### Sample population

**Fig. 1:**
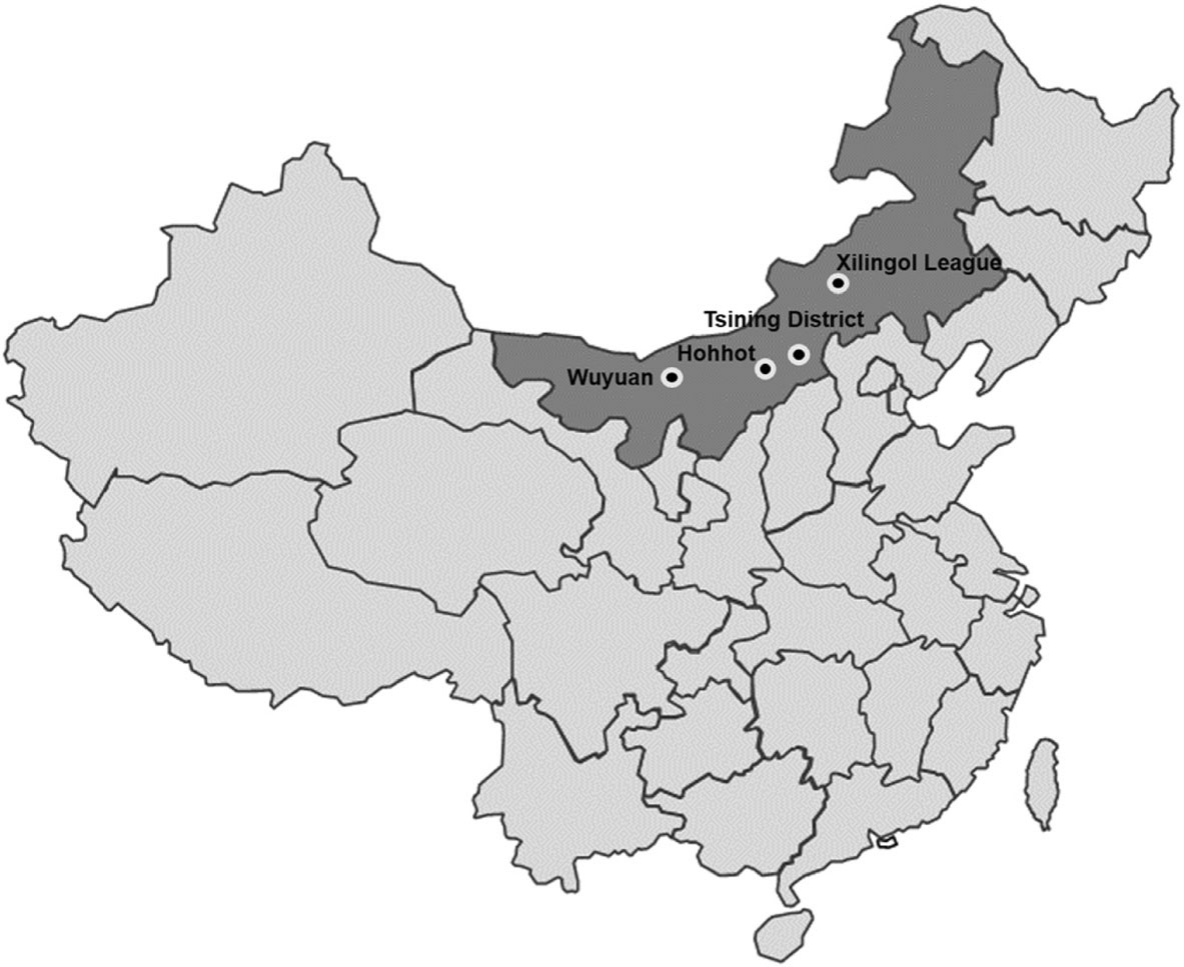
The map of China with four survey sites (Artificial drawing created by author YW)

In this cross-sectional study, stratified sampling was conducted according to level of urbanization, and four survey sites were enrolled (Table 1 and Fig. 1): Hohhot, a large city located at 40.83°N that has short sunshine duration and is little affected by sandstorms; Tsining District, a midsize city and commercial trade zone located at 41.03°N (high altitude), has longer sunshine duration and is known for its dry, windy weather and sandstorms; Wuyuan, a county seat located at 40.10°N with the longest sunshine duration among the survey sites; and Xilingol League, a pastoral area located at 44.58°N, which is at the lowest altitude and which is predominantly grassland with sufficient sunshine and precipitation. In these cities and counties, we randomly selected different districts in cities and rural townships. The samples were stratified according to the sex and age distribution of the Inner Mongolian population in the China Population Census 2010. We set the proportion of Han and Mongolian participants according to the proportions of the local population. This study included only residents who had lived in the area for more than one year and excluded patients with mental illness by self-reported, pregnant women, and active military personnel. Only residents whose ethnicity was the same as their parents’ were enrolled in the study; in other words, Han participants had Han parents, and Mongolian participants had Mongolian parents. Out of 3468 eligible residents, 3185 underwent ophthalmological examinations, with an overall response rate of 91.84%. We finally included 2651 people who were 30 years of age or older. This study is based on the principles of the Declaration of Helsinki. Ethical approval was granted by the bioethical committee of the Institute of Basic Medical Sciences, the Chinese Academy of Medical Sciences.

**Table 1:** Climatic conditions of the four survey sites

	Mean Elevation (meters)	Mean Annual Precipitation (millimeters)	Annual Sunshine Duration (hours)
Wuyuan	1042	170	3263
Hohhot	1040	400	1600
Tsining District	1419	384	3130
Xilingol League	1000	295	3000

### Physical examinations, data collection and stratification standard

The data collection was conducted in the field from July 2014 to August 2014. A team of three ophthalmologists, medical workers from general hospitals in Beijing and administrative personnel from the region carried out the data collection. Trained counsellors asked each participant questions and presented questionnaires to collect data, including information about age, sex, ethnic group, birthplace, residence (urban or rural), occupation (worker, farmer, management, service and sales, technical work, student, housework, etc.; agricultural work was classified as an outdoor occupation, while the other occupations were considered to be indoor occupations), education level (elementary or lower, middle school to high school, and university or higher), level of exercise [14] (light, moderate or heavy), history of hypertension and diabetes, and lifestyle (e.g., smoking and alcohol consumption). Smoking was divided into two categories: never-smokers and ever-smokers (including current smokers and former smokers). Similarly, alcohol consumption was classified into never-drinkers and ever-drinkers (including current drinkers and former drinkers). The systolic and diastolic blood pressure of the participants was measured three times using an electronic automatic blood pressure monitor (HEM-907, Omron, Japan). The average was used as the mean blood pressure. Height was measured by using a fixed stadiometer. Body weight and body fat percentage (BF%) were measured by bioelectrical impedance analysis (BC-420, Tanita, Japan). BMI (Body mass index) was calculated as weight in kilograms divided by height in metres squared (kg/m^2^).

### Ophthalmologic examination

Ophthalmologic examinations included visual acuity at 4 m (EDTRS, Wehen Co., Ltd., Guangzhou, China). Data related to refraction, such as corneal curvature radius, were measured with an auto ref-keratometer (ARK- 510A, Nidek Co., Ltd., Tokyo, Japan). Astigmatism was defined as cylinder value<− 0.50 D. We used a portable hand-held slit-lamp to examine the anterior segment of the eye (KJ5S2, Suzhou Kangjie Medical Co., Ltd., Jiangsu, China).

### Diagnostic criteria and grading standard of Pterygium

Pterygium (in either eye) was defined as a raised fibrovascular tissue encroaching through the limbus into the cornea or a history of pterygium excision. The examination of pterygium was performed by ophthalmologists with portable slit lamp. The grading was based on the location of pterygium head under standard lighting conditions [4, 10]. Grade 1: at the limbus. Grade 2: between the limbus and the undilated pupil margin. Grade 3: within the pupil margin. Grade 4: beyond the pupil margin. If bilateral pterygium was diagnosed, the higher grade eye was counted.

### Statistical analysis

All statistical analyses were performed using the SPSS software program (Statistical Package for Social Sciences Inc., Chicago, IL, USA, version 21.0.0.0). Figures were created with the GraphPad Prism 7.0 software program. We performed a chi-square test using the pterygium growth site and unilateral and bilateral as variables to calculate the prevalence of pterygium and calculated the age-adjusted prevalence after referring to the China Population Census 2010. Independent sample t-test and chi-square test were used to analyse the demographic characteristics of the Han and Mongolian participants and the grades of pterygium. The univariate analysis between presence of pterygium and factors was performed at first. Subsequently, we performed interaction terms in logistic regression to evaluate the factors which may cause variance inflation. The multivariate regression analysis model was used to assess possible risk factors or protective factors for pterygium afterward. Ultimately, we used sex and ethnicity as subgroups to further analyse relevant factors in a multivariate regression analysis.

## Results

### Prevalence and demographic characteristics

The final study population included 2651 participants of 30 years of age or older. Among these, there were 1043 (39.3%) men and 1608 women (60.7%) and 1910 Han Chinese (72%) and 741 Mongolian (28%) adults (Table 2). The average age of the participants was 48.93 ± 11.06 years (range 30–79 years).

**Table 2:** Characteristics of the Han and Mongolian participants

	Han	Mongolian	*P* value	
	*N* = 1910	*N* = 741		
Age, y	49.06 ± 11.15	48.59 ± 10.84	0.324	
Sex, *n*%			0.967	
Male	751(39.3)	292(39.4)		
Female	1159(60.7)	499(60.6)		
Height, cm	162.51 ± 8.08	162.80 ± 8.32	0.396	
Weight, kg	65.84 ± 12.02	67.97 ± 12.90	<0.001	***
BMI level			<0.001	***
<24	815(43.2%)	243(33.2%)		
24–27.9	725(38.5%)	306(41.9%)		
≥ 28	345(18.3%)	182(24.9%)		
Body fat percentage, *n*%	29.58 ± 7.70	30.99 ± 7.78	<0.001	***
Serum total cholesterol, mmol/L				
< 2.26	1(0.1%)	1(0.1%)	0.487	
≥ 2.26	1907(99.9%)	739(99.9%)		
Triglycerides, mmol/L				
< 6.22	1450(76.0%)	583(78.8)	0.127	
≥ 6.22	458(24.0%)	157(21.2%)		
Low-density lipoprotein cholesterol, mmol/L			0.009	**
< 4.14	383(20.1%)	116(15.7%)		
≥ 4.14	1525(79.9%)	624(84.3%)		
High-density lipoprotein cholesterol, mmol/L			0.228	
≤ 1.04	18(0.9%)	11(1.5%)		
> 1.04	1890(99.1%)	729(98.5%)		
Birthplace, n%			<0.001	***
Urban	853(44.7)	182(24.6)		
Rural	1057(55.3)	559(75.4)		
Residence, n%			<0.001	***
Urban	1772(92.8)	651(87.9)		
Rural	138(7.2)	90(12.1)		
Education, n%			<0.001	***
Elementary or lower	304(15.9)	93(12.6)		
Middle and high school	1106(57.9)	296(39.9)		
University or higher	500(26.2)	352(47.5)		
Occupation, *n*%			<0.001	***
Indoor	1767(92.5)	652(88.0)		
Outdoor	143(7.5)	89(12.0)		
Exercise level, n%			0.464	
Light	377(19.7)	112(15.1)		
Moderate	278(14.6)	67(9.0)		
Heavy	21(1.1)	5(0.7)		
Hypertension, *n*%	597(31.3)	268(36.2)	0.024	*
Systolic pressure, *n*%	352(18.4)	149(20.1)	0.322	
Diastolic pressure, *n*%	305(16.0)	151(20.4)	0.007	**
Diabetes, *n*%	98(5.1)	37(5.0)	0.136	
Alcohol, *n*%			<0.001	***
Never	1154(60.4)	369(49.8)		
Ever	756(39.6)	372(50.2)		
Smoking status, n%			0.8	
Never	1310(68.6)	512(69.1)		
Ever	600(31.4)	229(30.9)		
Survey sites			<0.001	***
Wuyuan	593(31.0)	199(26.9)		
Hohhot	393(20.6)	89(12.0)		
Tsining District	680(35.6)	31(4.2)		
Xilingol League	244(12.8)	422(57.0)		
Time in rural, d	4452.76 ± 5643.96	5954.18 ± 5974.69	<0.001	***

*P < 0.05, **P < 0.01, ***P < 0.001

The prevalence of pterygium in this study was 6.4% (169/2651, 95% CI 5.5–7.3), and the age-adjusted prevalence rate of pterygium was 6.38% among people aged 30 years and older. We noted that the prevalence increased with age (*P* < 0.001), which was increased by 1.36 times for every 10 years of age (95% CI = 1.23–1.49).

The incidence of unilateral pterygium was higher than that of bilateral pterygium (*P* < 0.001). We found a significant difference in prevalence between the right and left eyes (*P* < 0.001); specifically, 54 cases (43.0%) involved the right eye and 73 cases (57.0%) involved the left eye. Regarding the location of the disease, in the majority of cases, the pterygium was located on the nasal side (*n* = 38; 1.2%). In 4 cases (0.1%) the pterygium was located on the temporal side. There were no cases with pterygium on both sides.

### Grading analysis of Pterygium

**Fig. 2:**
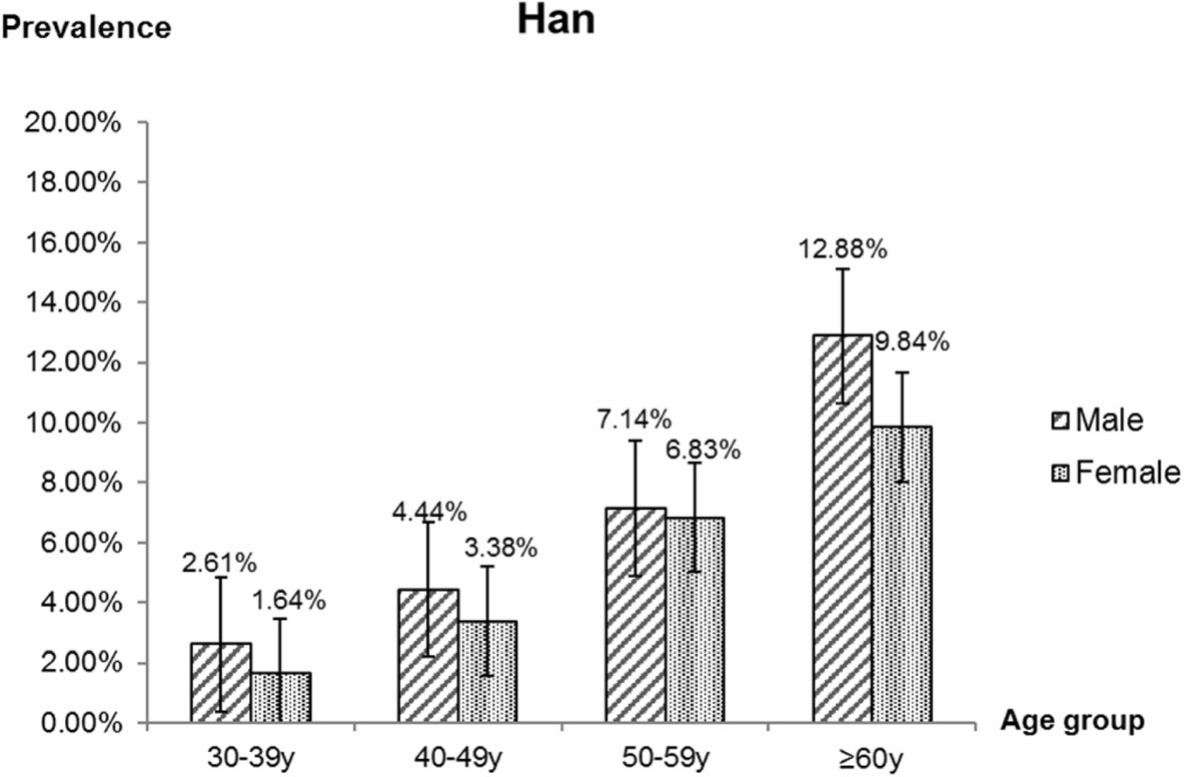
Age distribution of Han male and female with pterygium grade 2

**Fig. 3:**
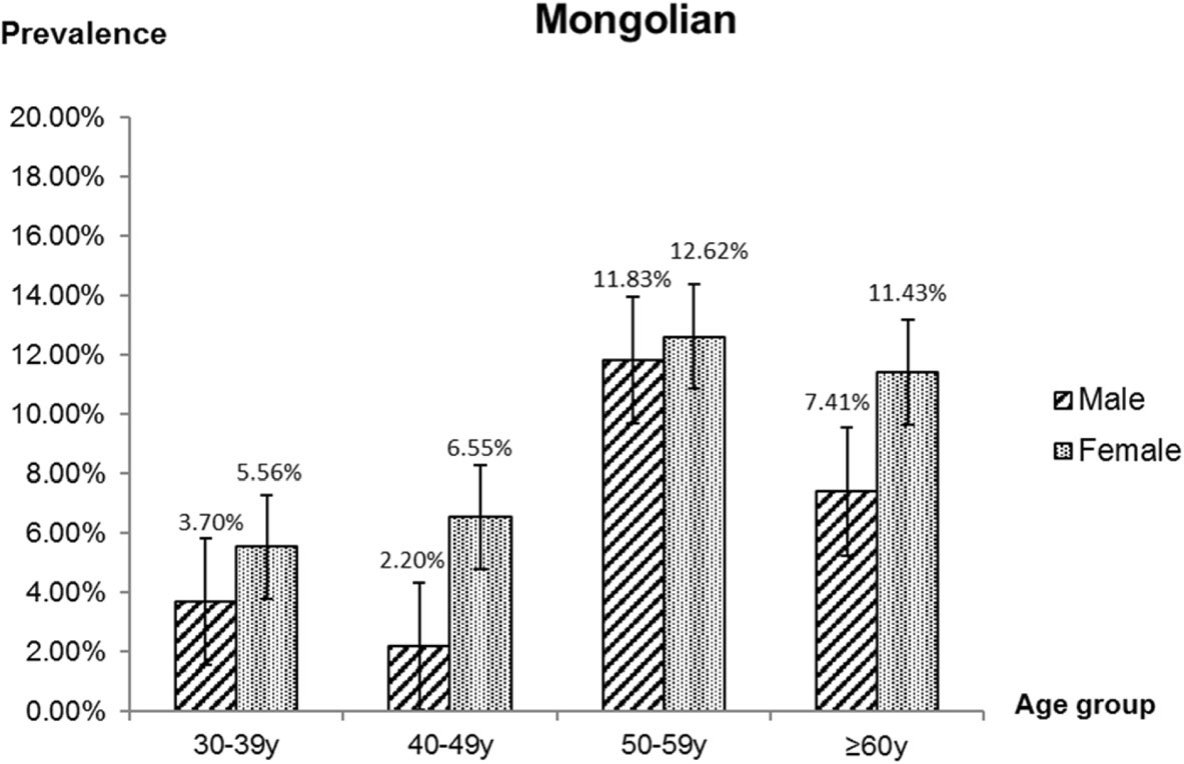
Age distribution of Mongolian male and female with pterygium grade 2

Regarding the grading of pterygium, 5 (0.2%) cases were classified as were grade 1, 162 (6.2%) were classified as grade 2, and no cases were classified as grade 3 or 4. In general, grade 2 was the most common grade among the two ethnic groups. As Figs. 2 and 3 shown, the Grade 2 were mainly concentrated in Mongolian adults of 50–59 years of age.

### Multivariate logistic regression results

Frist of all, we performed the interaction terms to evaluate the relationships between time in rural, occupation and education level (Tables 3, 4 and 5), the relationships between BMI, Low-density lipoprotein cholesterol (LDLC) and Body fat percentage (Tables 6, 7 and 8). Since there was no participant who had university or higher education level worked outdoor, we combined the “middle school to high school” and “university or higher” together as a new group, which called “middle school or higher”. We found that BMI<24–27.9 * LDLC≥4.14 had statistical difference (β = − 1.17, OR = 0.31, 95% CI: 0.10–0.96, *P* = 0.041). The education level and occupation influence each other with statistical difference (β = 1.01, OR = 2.76, 95% CI: 1.82–4.18, *P* < 0.001). At the same time, the occupation and time in rural could influence each other with statistical difference (time in rural> 30 years*outdoor: β = − 1.67, OR = 0.19, 95% CI: 0.07–0.19, *P* = 0.001). The multivariate logistic regression model was based on a univariate analysis and study settings; with the following factors included in the final calculation: ethnicity, gender, age group, smoking status, education level, occupation, BMI, body fat percentage (BF%), low density lipoprotein cholesterol, survey site and time spent in rural areas. The results showed that having an outdoor occupation (OR 1.78, 95% CI: 1.09–2.90, *P* = 0.020), living in rural areas for more than 30 years (OR 1.98, 95% CI: 1.29–3.03, *P* = 0.002), and being 50–59 years old (OR 2.86, 95% CI: 1.59–5.16, *P* < 0.001) or ≥ 60 years old (OR 2.79, 95% CI: 1.48–5.26, *P* = 0.001) were risk factors for pterygium. Living near an urban survey site such as Hohhot (OR 0.44, 95% CI: 0.26–0.76, *P* = 0.003) or Tsining District (OR 0.30, 95% CI: 0.17–0.52, *P* < 0.001) were protective factors. We found that pterygium had no correlation with sex, ethnicity, BF%, low density lipoprotein cholesterol or smoking status. The results of the logistic regression analysis are presented in Table 9.

**Table 3:**
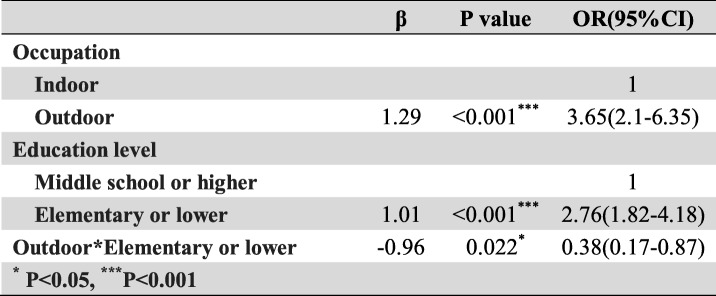
The interaction between occupation and education level

**Table 4:**
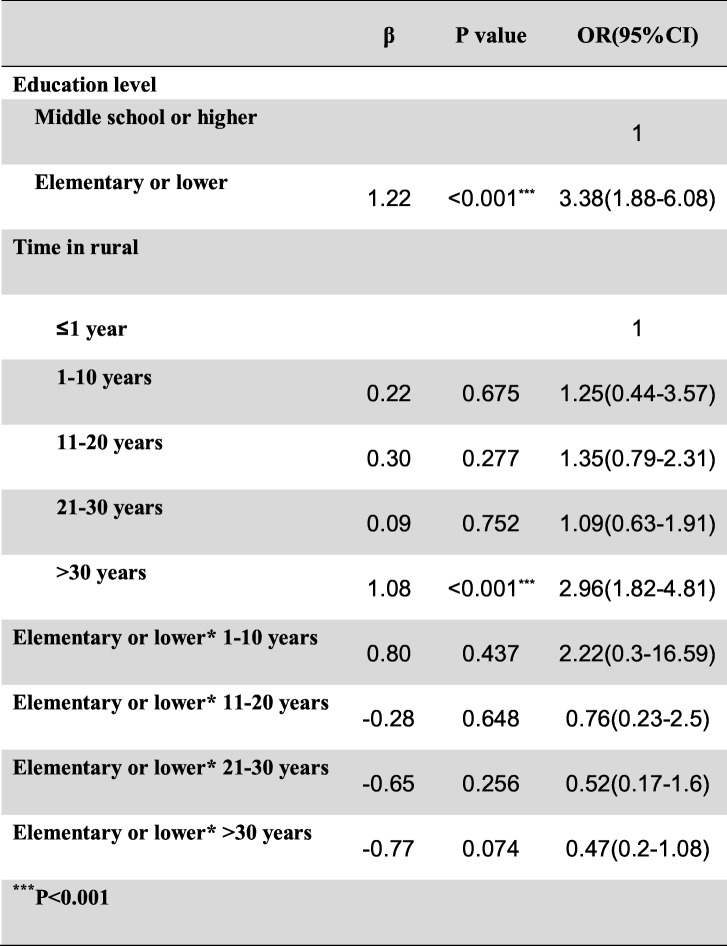
The interaction between education level and time in rural

**Table 5:**
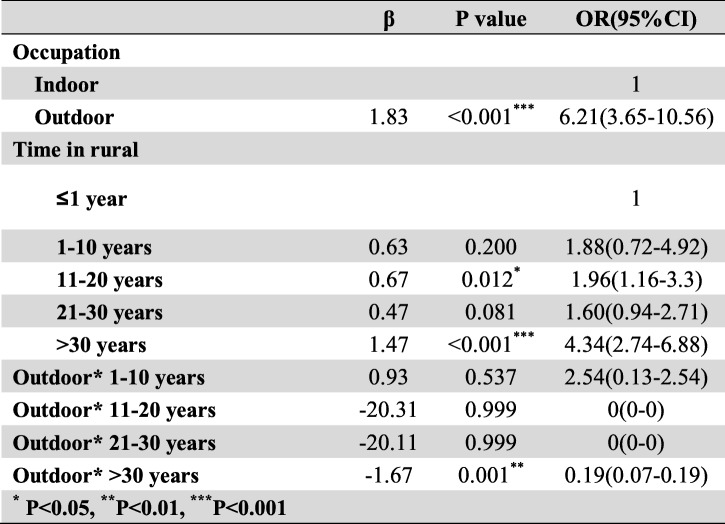
The interaction between occupation and time in rural

**Table 6:**
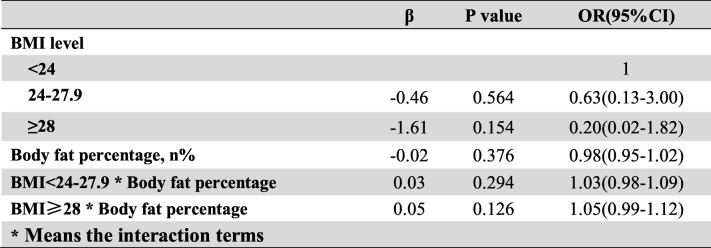
The interaction between BMI level and body fat percentage

**Table 7:**
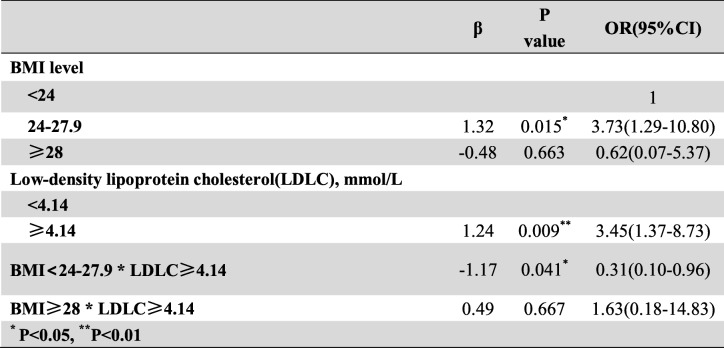
The interaction between BMI level and low-density lipoprotein cholesterol

**Table 8:**
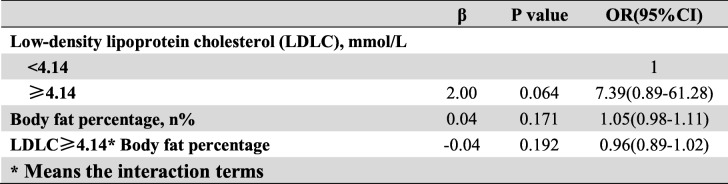
The interaction between low-density lipoprotein cholesterol and body fat percentage

**Table 9:** Multivariate logistic regression analysis for factors associated with pterygium

	OR(95% CI)	*P* Value	
Age, y			
30–39	1		
40–49	1.22(0.66–2.25)	0.518	
50–59	2.86(1.59–5.16)	< 0.001	***
≥ 60	2.79(1.48–5.26)	0.001	**
P for trend		< 0.001	
Sex			
Male	1		
Female	0.62(0.28–1.38)	0.240	
Race			
Han	1		
Mongolian	0.92(0.63–1.35)	0.674	
Survey sites			
Wuyuan	1		
Hohhot	0.44(0.26–0.76)	0.003	**
Tsining District	0.30(0.17–0.52)	< 0.001	***
Xilingol League	1.02(0.67–1.55)	0.941	
P for trend		< 0.001	
Time in rural			
≤ 1 year	1		
1–10 years	1.47(0.59–3.62)	0.405	
11–20 years	1.48(0.89–2.47)	0.135	
21–30 years	0.95(0.56–1.60)	0.833	
> 30 years	1.98(1.29–3.03)	0.002	**
P for trend		0.015	
BMI level			
<24	1		
24–27.9	0.88(0.52–1.46)	0.611	
≥ 28	0.45(0.19–1.03)	0.058	
P for trend		0.060	
Body fat percentage, n%	1.04(0.99–1.10)	0.132	
Low-density lipoprotein cholesterol (LDLC), mmol/L			
< 4.14	1		
≥ 4.14	1.51(0.90–2.54)	0.123	
Education level			
Elementary or lower	1		
Middle school or higher	0.70(0.45–1.09)	0.114	
Occupation			
Indoor	1		
Outdoor	1.78(1.09–2.90)	0.020	*
Smoking status			
Never	1		
Ever	1.09(0.69–1.72)	0.719	

* *P* < 0.05, **P < 0.01, ***P < 0.001

### Subgroup analysis of Pterygium

**Fig. 4:**
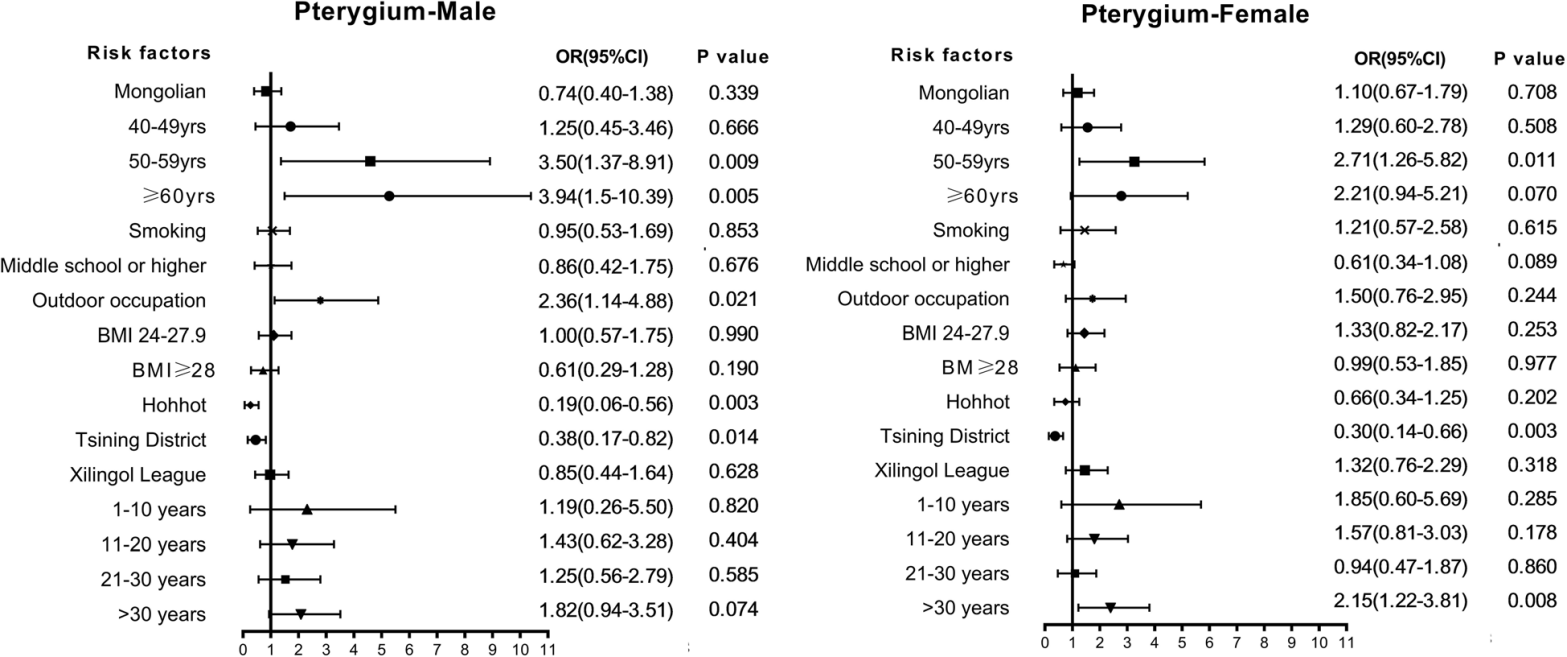
Multivariate analysis of factors associated with pterygium in male and female

**Fig. 5:**
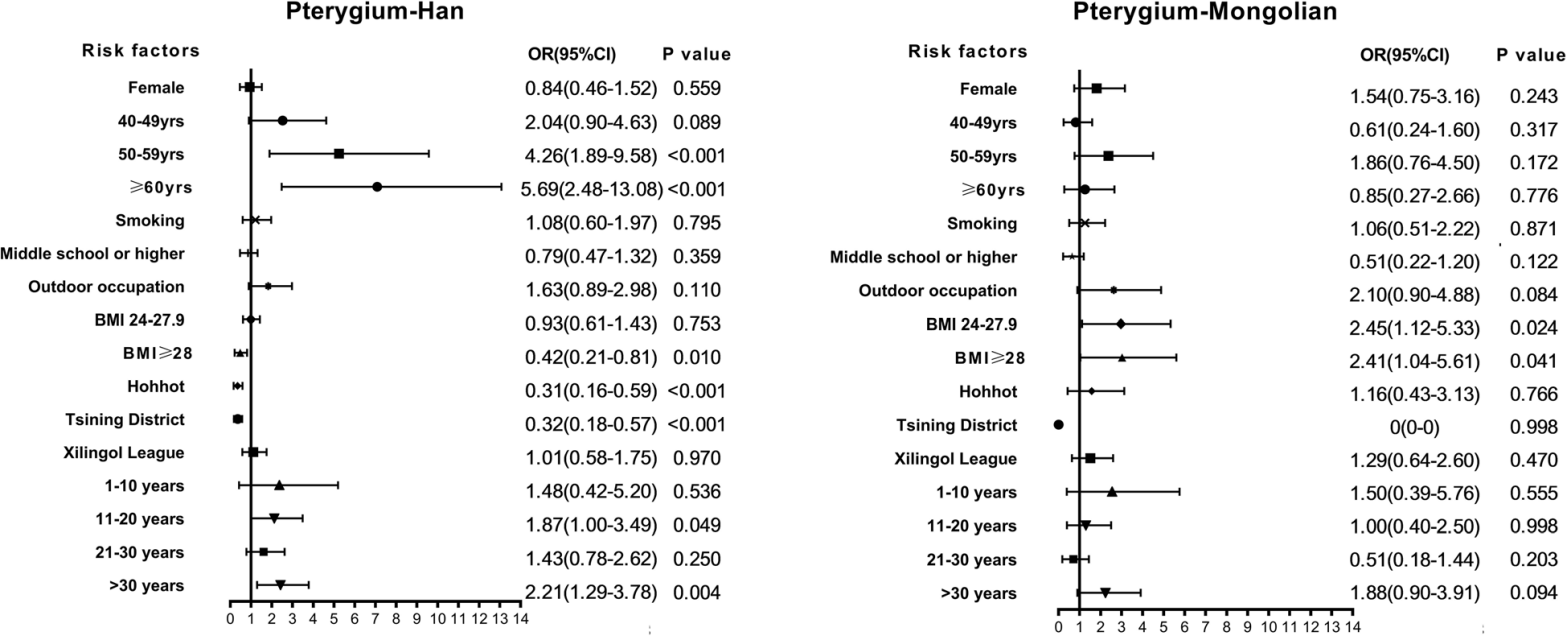
Multivariate analysis of factors associated with pterygium in people of Han or Mongolian ethnicity

We also performed a subgroup logistic regression analysis using ethnicity and sex as the classification criteria (Figs. 4 and 5).

From the results of the ethnic subgroups, being older and having lived longer in rural residence areas risk factors among male and female participants (50-59 yrs. Male OR = 3.50, 95% CI: 1.37–8.91, *P* = 0.009; > 60 yrs. Male OR = 3.94, 95% CI: 1.5–10.39,*P* = 0.005; 50-59 yrs. Female OR = 2.71, 95%CI: 1.26–5.82, *P* = 0.011;. Residing in Hohhot and Tsining District were protective factors (Male in Hohhot OR = 0.19, 95% CI: 0.06–0.56, *P* = 0.003; Male in Tsining District OR = 0.38, 95%CI: 0.17–0.82, *P* = 0.014; Female in Tsining District OR = 0.30, 95%CI: 0.14–0.66, P = 0.003). In the analysis of male subjects alone, having an outdoor occupation was a risk factor for pterygium (OR = 2.36, 95% CI: 1.14–4.88, *P* = 0.021). When referring to female subjects, living in rural for more than 30 years was a risk factor (OR = 2.15, 95% CI: 1.22–3.81, *P* = 0.008).

In the ethnic subgroup analysis, being older and having lived in a rural area for 11–20 years or > 30 years were risk factors for pterygium in Han adults (50-59 yrs. OR = 4.26, 95% CI: 1.89–9.58, *P* < 0.001; > 60 yrs. OR = 5.69, 95% CI: 2.48–13.08, *P* < 0.001; time in rural 11-20 yrs. OR = 1.87, 95% CI: 1.00–3.49, *P* = 0.049; time in rural > 30 yrs. OR = 2.21, 95% CI: 1.29–3.78, *P* = 0.004). A high education level, BMI ≥ 28, and residing in Hohhot and Tsining District were protective factors in Han participants (Hohhot OR = 0.31, 95% CI: 0.16–0.59, *P* < 0.001; Tsining District OR = 0.32, 95%CI: 0.18–0.57, *P* < 0.001). BMI ≥ 24 was a major risk factor for pterygium in the Mongolian adults (BMI = 24–27.9 OR = 2.45, 95% CI: 1.12–5.33, *P* = 0.024; BMI ≥ 28 OR = 2.41, 95% CI: 1.04–5.61, *P* = 0.041). BMI ≥ 28 was a risk factor in Han adults as well (OR = 0.42, 95% CI: 0.21–0.81, *P* = 0.010). None of the Mongolian participants in Tsining District suffered from pterygium.

## Discussion

This was the first cross-sectional study of Han and Mongolian adults in the Inner Mongolia Autonomous Region of China. A stratified sampling method was used to include 3185 participants who underwent ophthalmologic examination at the four survey sites. The mean age was 48.93 ± 11.06 years, and the overall prevalence after age adjustment was 6.38%. Pterygium most frequently occurred on the nasal side (38/2651, 1.4%), and the most common grade was grade 2 (162/2651, 6.1%). Having an outdoor occupation, living in a rural area for > 30 years, and being > 50 years of age were risk factors for pterygium. Having a university or higher education level and living near an urban survey site (Hohhot and Tsining District) were protective factors.

Numerous epidemiological surveys have confirmed that aging is an important risk factor for pterygium [4, 10, 15]. The prevalence of pterygium in people over 80 years of age has been found to be as high as 19.5% [1]. Similar to the results of the above studies, our results showed that participants over 50 years of age had a significantly increased risk of pterygium.

In a 2004 study of the relationship between the onset of pterygium and UV exposure time in Hainan Province, China [16], a research group confirmed that length of pterygium was positively correlated with UV exposure time. The study participants were divided into three groups:youth, middle-aged, and old age. The length of pterygium was positively correlated with UV exposure time, with those in the old group having had pterygium the longest. Accordingly, we hypothesize that the increase in the prevalence of pterygium in people over 50 years of age may be related to an increase in the cumulative UV exposure time.

There is currently no consistent conclusion on the relationship between sex and pterygium. Many studies, such as those in China, India, Japan, Singapore, Iran and Ethiopia [9, 11, 17–21], have found that being a man was a risk factor for pterygium. Nevertheless, the study of Dali and the Tibet Autonomous Region of China confirmed that being a woman was risk factor; this may be related to the social division of men and women in the cultures of different regions. In most parts of Asia and Africa, men represent the main labour force in the family, women spend most of their time doing indoor housework. In recent years, with the process of non-agriculturalization in western China, the social division of labour among Inner Mongolian farmers and herdsmen has changed [22, 23]. More men choose to go to work in large cities, while women stay in rural areas for agricultural activities or livestock farming. During the busy farming season, men return to the countryside to participate in agricultural work. Thus, in general, men and women spend roughly the same amount of time doing outdoor work. Our data analysis also confirmed that there was no significant differences in the prevalence of pterygium between men and women, which is similar to the results of a study conducted in Spain in 2011 (prevalence: 4.8% in men, 6.5% in women, *P* = 0.346) [24].

Previous multiethnic studies on pterygium have found differences in the prevalence among ethnicities; for example, the prevalence of pterygium was significantly increased among people of Han ethnicity in Xinjiang and among people of Yi ethnicity in Yunnan [4, 10]. Our study found no significant difference in the prevalence of pterygium between Han and Mongolian people (5.8% vs 7.8%, *P* = 0.06). There was no significant association between ethnicity and pterygium after adjustment for age and sex. According to the China Population Census 2000, the intermarriage rates between Han and Uygur, Han and Yi were 0.62, 16.29% respectively, which were much lower than Han and Mongolian (37.49%). Mongolian ethnicity is a large ethnic group in Inner Mongolia and used to have mixed multi-ethnic regions with Han ethnicity since Yuan dynasty [25, 26]. According to the China Population Census 2000, the mixed ethnic household rate in Inner Mongolia was 11.70%. Meanwhile, the Uygur ethnicity is Caucasian, Mongolian and Han ethnicity both belong to Mongolian. We hypothesised that the Han and Mongolian ethnicities had similar lifestyle.

The multivariate analysis in the present study demonstrated that living in towns such as Hohhot and Tsining District were protective factors for pterygium. This may be related to their urbanization, residents’ living habits and the geographical environment, in which the sunshineduration is short and there is little sandy wind. In a study in Jordan in 2004 [27], living in a dry, dusty environment with long-term exposure to large amounts of particles was identified as a risk factor for pterygium. Although Tsining District has an extremely dry climate and sandy wind, during the epidemiological investigation, we observed that the local residents paid attention to self-protection and went outside wearing sunglasses and hats. Interestingly, after pooling the influence of environmental and social factors on pterygium, the results of multivariate analysis showed that living in Tsining District was a protective factor. This reminds us that in a dry and dusty environment, using more protective measures and avoiding long-term exposure to ultraviolet light will greatly reduce the occurrence of pterygium. Protective factors and risk factors are influenced by culture, geography and health awareness.

Our results confirmed a positive correlation between the incidence of pterygium and education level. The prevalence of pterygium in university or higher education level was lower than that of middle or high school education level (3.4% VS 6.3%). This is similar to the results of a multiethnic study in Malaysiaand a Latin American study in 2009 [28, 29]. In the present study, there was no people who had university or higher education level had outdoor occupation. We hypothesize that because people with higher education levels have a higher likelihood of working indoors, they have less exposure to sunlight and would therefore be exposed to ultraviolet light for a shorter period of time.

Outdoor activities, including surfing and fishing have been reported to be risk factors for pterygium in previous studies [8, 30]. At the same time, high-intensity exposure to ultraviolet light during youth increases the risk of pterygium. A case-control study in Brisbane in 1992 and a study on Norfolk Island in 2013 suggested that the cumulative duration of UV exposure had a greater impact on pterygium [31, 32]. Consistent with these results, we found that living in rural areas longer than 30 years and an outdoor occupation were risk factors for pterygium.

We usually use BMI to quantitatively analyse obesity, but the effect of high BMI on pterygium is still inconclusive [33, 34]. In our subgroup analysis, BMI ≥ 28 was a protective factor for Han (OR 0.42, 95% CI 0.21–0.81, *P* = 0.010), while it was a risk factor for Mongolian (OR 2.41, 95% CI 1.04–5.61, *P* = 0.041). However, BMI is not the only index used to evaluate obesity. In recent years, the body fat percentage (BF%), which assesses the fat mass more effectively than the BMI, has also been used to evaluate the degree of obesity [35, 36]. Previous studies have found that the Chinese have lower BMI and higher BF% [37]. There were great differences in the diet and living habits of the Han and Mongolian ethnicities [38, 39]. In comparison to the Han ethnicity, Mongolians had a higher meat and salt intake and a lower intake of fruits and vegetables, which meant that Mongolians ingested more fat and protein [40]. It is well recognized that high-fat diets can increase the oxidative stress level in the body, and the conclusion that oxidative stress is caused by high-protein diets remains controversial [41, 42]. The oxidative stress caused by obesity may have an effect on pterygium [43]. Hence, we used participants’ BF% to more accurately assess obesity. The BF% of the Han ethnicity was 29.58 ± 7.70%, and the BF% of the Mongolian ethnicity was 30.99 ± 7.78%, which represented a significant difference (*P* < 0.001). Therefore, we believe that although the BMI value of both ethnic groups were elevated, the different dietary structures lead to different fat content and therefore different degrees of oxidative stress. However, the effects of systemic oxidative stress on the ocular surface need to be confirmed by further studies.

The association between smoking and pterygium has been controversial. Certain studies have suggested that smoking is a risk factor for pterygium [44], but a meta-analysis in 2014 and a survey in Israel in 2016 showed that smoking had a protective effect [45, 46]. The biological impact of smoking on pterygium remains unclear. Some researchers have speculated that smoking could inhibit the expression of pro-inflammatory cytokines, reduce ocular surface inflammation, and inhibit vasoconstriction through various receptors [47, 48]. In our study, we found that smoking was not a factor related to pterygium in people of Han or Mongolian ethnicity.

The present study was associated with some limitations. First, information concerning aspects such as medical history and living habits was collected by a questionnaire, which allowed for a recall bias. Second, we did not quantitatively measure the intensity or duration of UV exposure. Finally, as this was a cross-sectional study, we were unable to determine the causal relationship between these factors and pterygium. The next step should be undertake a cohort study.

## Conclusions

This study investigated the prevalence of pterygium and associated factors in Han and Mongolian adults at four survey sites in Inner Mongolia, China. The overall prevalence of pterygium was 6.4%. The outdoor occupation, old age (≥50 yrs) and more time spent in rural areas (> 30 yrs) were risk factors for pterygium. Town as a survey site (Hohhot and Tsining District) was a protective factor for pterygium. Based on the present study, we should focus on the education of outdoor workers in rural and encourage them to take protective measures such as wearing hats and sunglasses.

## Data Availability

Not applicable.

## Abbreviations

BF%: Body fat percentage
BMI: Body mass index
CNHS: China National Health Survey
LDLC: Low-density lipoprotein cholesterol
